# Protein Dynamics: From Molecules, to Interactions, to Biology

**DOI:** 10.3390/ijms10031360

**Published:** 2009-03-20

**Authors:** Martin Gruebele

**Affiliations:** Center for Biophysics and Computational Biology, Department of Chemistry, Department of Physics, and Beckman Institute for Advanced Science and Technology, 600 South Mathews Ave., Urbana, IL 61801, USA; E-Mail: gruebele@scs.uiuc.edu

**Keywords:** Downhill folding, evolution, protein function

## Abstract

Proteins have a remarkably rich diversity of dynamical behaviors, and the articles in this issue of the *International Journal of Molecular Sciences* are a testament to that fact. From the picosecond motions of single sidechains probed by NMR or fluorescence spectroscopy, to aggregation processes at interfaces that take months, all time scales play a role. Proteins are functional molecules, so by their nature they always interact with their environment. This environment includes water, other biomolecules, or larger cellular structures. In a sense, it also includes the protein molecule itself: proteins are large enough to fold and interact with themselves. These interactions have been honed by evolution to produce behaviors completely different from those of random polymers.

## 1. Introduction

This issue of *IJMS* explores many of the aspects of protein dynamics, from folding to protein-protein interactions to crowding and interaction with surfaces. The methods applied range from sophisticated computational simulation to stopped-flow and chromatography. Many of the articles are designed to provide an overview of a specific area, along with some new thoughts, while others present research results advancing further our knowledge of proteins.

In this essay introducing the issue, I will discuss some of the general threads that pervade the contributions, and highlight some of the areas and results discussed in the contributions. I begin with a brief introduction to proteins and their dynamics, and from there gradually move to proteins in biophysical studies, such as folding, to proteins interacting with other biomolecules, to proteins in their *in vivo* environments fulfilling their function.

## 2. Proteins

Proteins are biologically evolved polymers constituted of about twenty chiral amino acids (the exact number depends on whether one wants to count post-translational modification, selenomethionine, certain rare amino acids in select organisms, etc.). We generally distinguish proteins from peptides in that they have both secondary structure and a hydrophobic core. The hydrophobic core segregates some side chains into the interior of the protein structure, creating a compact folded state. Hydrophobicity is an entropic driving force that increases the affinity for certain amino acid side chains for the interior of the protein. Although many details are still under active investigation, solvent entropy is a key factor in creating this driving force [1]. The solvent and the protein are thus an inseparable entity.

The term protein is also applied more widely: many DNA sequences do not code for amino acid sequences that fold *in vitro*, yet they have function *in vivo*. Such disordered polypeptides are also considered proteins [2]. In fact, it is an open question how many of these would fold *in vivo*, either because of non-specific crowding in the very dense environment inside a cell (≈ 200 mg/mL of biomaterial), or through specific folding-binding interactions. Models have shown that folding upon binding can enhance biological activity of proteins [3].

Because there are only roughly 20 natural amino acids, proteins are not perfectly packed, even when they fold. An infinite variety of sub-divisible shapes would be required to achieve perfect packing. Thus the often-heard notion that side chains fit together ‘like pieces of a puzzle’ is strictly speaking wrong, as is the notion that proteins are three-dimensional objects. Computational analysis has shown that proteins have fractal structure with a dimension less than three [4], and there is a hierarchical size distribution of cavities in their interior. In a sense, this brings proteins in line with other biological objects, which are always based on folded surfaces, and therefore objects of dimension greater than two but less than three. Take for example the endoplasmic reticulum, the packing of the mammalian brain (really a wrinkled sheet), or the structure of the lung; like a sheet of paper that has been wadded up into a ball, they are neither two-dimensional nor truly three-dimensional.

Misfitting of sidechains during packing, or non-native interactions at the surfaces of proteins that interact with their environment, can lead to frustration. There is a best – but still imperfect – fit, and then there are less optimal alternatives that still provide a measure of fit. Evolution minimizes such frustration, leading to consistency of the interactions on all relevant length scales [5,6]. Simulators reap the rewards of this consistency: proteins fold and interact remarkably quickly over remarkably small barriers, and make remarkably few errors while doing so.

Good is not perfect however. While the idea that proteins must be assembled by machinery in the cell was abandoned after Anfinsen’s *in vitro* proof of reversible folding [7], the larger a protein gets, the more error-prone the process becomes. Naturally, solutions have evolved to cope with this problem. The simplest is to limit the size of proteins and folding domains. Analysis of the manifold structures in the Protein Data Bank reveals that there is an upper size limit to autonomously folding protein domains. It lies at about 200 residues. Much larger proteins exist, but they are composed of multiple such domains that interact through their surfaces and limited linkage. While domains can interact strongly during function and even folding, they are also highly autonomous. For example, the 415 residue enzyme phosphoglycerate kinase is composed of two domains connected by a linker. The domains interact somewhat during folding, and very much so during function. But if the protein is clipped into two domains, each folds on its own [8,9]. Thus nature switches from a ‘make a new fold’ to a ‘snap together folds as building blocks’ when proteins get larger. The second solution is cellular machinery that protects proteins from making errors during folding. The ribosome itself, heat shock proteins, GroEL and many other examples are known that act by binding to misfolded proteins to unfold them and give them another chance, or encapsulate proteins to provide an undisturbed environment for folding [10].

Consistency and minimal frustration have an interesting effect on the free energy landscape of the protein, but first I must briefly define this landscape. Protein dynamics at the most elementary level involves the vibration of every bond and rotation of every nearby water molecule, *in toto* tens of thousands of coordinates. If these coordinates were not correlated into a smaller number of collective coordinates, protein dynamics would be uninteresting: it would be like the random Brownian motion of many pollen grains. Instead, protein sequences evolved to introduce strong correlations among microscopic coordinates to build up a few functionally important collective coordinates, while other microscopic coordinates just average into the thermal noise. These emergent collective coordinates are what makes any biological function possible. When we write the energy in terms of only these key coordinates, we have a free energy landscape, instead of the full energy function of a protein. The free energy landscape weights these coordinates in two ways: Do they lower the energy of the protein and its interacting solvent and binding partners? Do they lead to more configurations, making certain protein structures statistically more likely? Both lowering the energy and increasing the entropy can lead to local minima in the free energy landscape. It is for this reason that unfolded proteins, or proteins not bound to their substrate, nonetheless can be at a low free energy: the sheer additional number of conformations in that case wins out over low energy of any given conformation [11].

**Figure 1.**
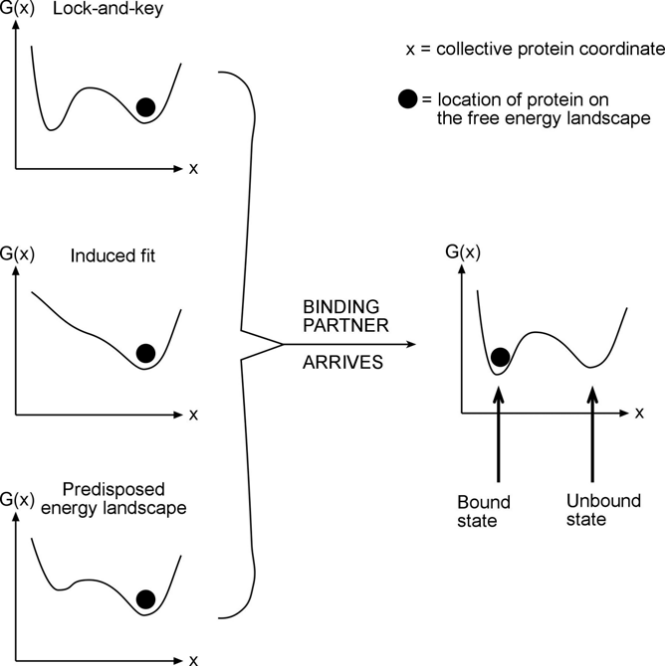
The concept of a ‘predisposed’ free energy landscape.

Now on to the effect of frustration on free energy surfaces. Proteins, to a remarkable degree, already have local minima built into their free energy landscapes where they need them. For example, a protein in an unbound state may be in a certain local minimum (Figure 1). When it binds, the question is: is the new minimum already there, fully developed – this would be a “lock and key” mechanism, or does the free energy landscape have to resculpt itself to create a new minimum from scratch – this would be an “induced fit” mechanism. The answer is: neither! Free energy surfaces usually have low-lying minima that are already conducive to the desired dynamics, but these minima will be lowered further in free energy through the actual interaction event.

Once the molecule interacting with the protein arrives, a driving force towards the desired state already exists, and is strengthened further by shifts in the relative free energy. Nowhere is this more evident than in folding itself: NMR studies of alpha-carbon ^13^C chemical shifts and many other experiments have shown that unfolded states have remarkably rich residual structure [12]. It would not be a great exaggeration to say that they are native states, but with somewhat randomized Ramachandran angles! The free energy landscape is predisposed for folding, and proteins never stray far from the native state. In terms of the energy funnel picture developed in the mid-90s [13,14], even unfolded states do not occupy the upper reaches of the funnel where completely random structures exist. The collective coordinates do not need to be twisted too much to get a protein to fold. This accounts for a remarkable observation about protein folding: it is a very fast process. Most organic molecule chemical reactions take days, or years, to cross the activation barrier at room temperature, yet even ‘slow’ proteins fold in less than a few hours. All this is thanks to the existence of a small number of collective coordinates, which are already nearly native-like. In this context, a statement such as ‘but your folding rate is just fast because your initial state is not a random coil’ are seen to be misguided. A protein cannot hope to sample in its lifetime of a few hours to weeks even a small fraction of the random configurations available to it in principle.

## 3. From Proteins, to Interactions, to Biology

I now discuss some of the ideas brought forth in this issue, in the general context of protein dynamics from internal dynamics to effects of the environment. There is not enough room to go over every paper, but hopefully the appetite of the reader will be wetted to read the many interesting contributions.

The article by Brooks nicely fits in with the points made above about minimal frustration. When doing computations, practitioners of the art frequently use “Go-potentials” (named after Nobuhiro Go, and the idea of consistent interactions [5]). Basically this means that any non-native interactions are removed from the free energy, and only the minima conducive to the desired process are left. One might expect such potentials to be “to god to be true” as they build in knowledge of the native state of a protein, but the rates and mechanisms found computational in “Go” calculations often match remarkably well with experiments. This emphasizes how highly evolved proteins are, and how unlike random heteropolymers.

I discussed above that proteins can fold rapidly because their unfolded states are predisposed for native structure. The paper by Pande and coworkers is the flip side of that coin: a human-designed beta sheet protein, found to fold fast experimentally [15], also does so in long time multi-trajectory molecular dynamics simulations, but may not form as extensive beta sheet structure as one might hope. This could be a case where the native state is close to the unfolded state, not *vice-versa*. The results also begs another question currently under intensive investigation: how good are the force fields? Can they even fold a protein to a native state? It is well known that different force fields have different errors in helical *vs* beta sheet hydrogen bonding, neglect three-body interactions etc. As a consequence, sometimes one gets the right result [16], sometimes one can end up folding a protein to a non-native structure that is a false global minima of the free energy [17]. The good news is that with many new ultrafast experimental folding studies appearing, there will be a whole new data base of results to allow tweaking of force fields to perfection [18,19]. The small molecule data and nanosecond calibrations are no longer sufficient when huge molecules need to be simulated over microseconds, milliseconds, or longer. It is much more important right now that failures of force fields be cataloged and analyzed, than successes, if the field is to move forward.

Computational tools like the above play an important role in moving the field of protein dynamics forward, and there are several more articles in that vein. For example, For example, Zacharias examines how biasing potentials in REMD can give us more information about the free energy surface and its local minima with less computer time. The idea of REMD (replica exchange molecular dynamics)[20] is to run many simulations of a protein at the same time, but at different environmental conditions (say, different temperatures). Then by switching proteins from one simulation to another, one can sample the free energy landscape more effectively. Zacharias adds to this the idea of biasing the surface to interrogate primarily the region of interest for a particular problem. Xi and coworkers examine the simulation literature for Trpzip2, a beta strand peptide that has become a standard test-bed for simulations and experiment.

**Figure 2.**
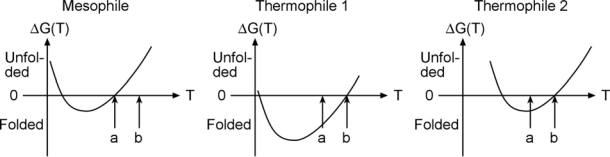
Thermophiles: stable everywhere or at high T only?

An experimental example of free energy tuning in the spririt of Figure 1 is the work by Takano and coworkers on folding of hyperthermophilic proteins. They show that many cases with extremely slow unfolding rates exist, so the native well is indeed tuned ‘deeper’ in order to make such proteins remain stable at high temperature. An interesting question in this context is whether the high-temperature stability arises because the free energy curve is shifted with temperature, or just lowered. Evidence exists for both scenarios in the literature (Figure 2).

As seen in the Figure, proteins denature at both high and low temperature (so called ‘cold denaturation’); somewhere in-between the free energy is lowest and the protein’s folded state is most stable. Some thermophiles achieve their high temperature stability simply by shifting the free energy curve to the right, so it intersects 0 free energy (equal stability of folded and unfolded state) at “b” instead of “a”. But others are genuinely more stable in an absolute sense, shifting the curve down in order to move melting from “a” to “b” (middle). The very slow unfolding data by Takano supports high native stability, and the middle scenario.

The middle scenario has an interesting consequence for downhill folding. Downhill folding is a process first postulated by energy landscape theory [13], and observed recently [21–23], whereby a protein is so stable that even its transition state for folding is at low free energy. Such a protein folds without a significant activation barrier, extremely rapidly (at most a few microseconds). We recently showed that what prevents many proteins from folding downhill, is that as one lowers the temperature from the melting point to make them more stable, they begin to cold denature before they can fold downhill [24]. But a thermophilic protein like the one in the middle of Figure 2 should be a perfect candidate for hyperstability, and hence downhill folding. Stability does not guarantee downhill folding because desolvation during folding can still create a large free energy barrier, but stability helps.

Desolvation is just one of the many ways a protein interacts with its environment. The article by Kinoshita discusses that water entropy, altered over significant distance scales from the protein, can make a large contribution to the folding free energy. Unlike a small molecule reaction, proteins cannot be treated as the ‘system’ with some small allowance for a solvation shell ‘perturbation.’ The solvation shell contributes a large fraction, perhaps the majority, of the free energy change. While NMR spectroscopy and X-ray crystallography have focused on the first solvation layer, or solvent within pockets in the protein, other techniques now also lend support to the notion of a far reaching dynamical affect of the protein on water, and vice versa. Work by Frauenfelder and coworkers shows that protein motions are slaved to dynamics of the solvation shell [25], while Terahertz spectroscopy measurements reveal that water is dynamically perturbed nanometers from the surface of a protein [26,27]. At 200 mg/mL density of biomolecules in a cell (the typical density of proteins is 1.4 g/cm^3^, 1-1/2 times that of water), that means no biological water is left within a cell.

Proteins interact with many other biomolecules in the cell. Cheung and coworkers review how protein shape becomes an important factor in stabilizing proteins that find themselves in a crowded environment full of carbohydrates (and other proteins, RNA, etc.). Sometimes these interactions enhance protein aggregation, sometimes they can prevent it. Kurganov and coworkers discuss how alpha crystallin can actually prevent aggregation by preventing the formation of larger clusters of proteins from interacting. Such clusters or ‘proto-aggregates’ are now implicated as cytotoxic, and may be the foundation of toxicity in many protein aggregation diseases, in lieu of, or in addition to, fibrils that eventually form. These diseases are omnipresent, and almost any protein can form aggregates, proto-fibrils, and eventually fibrils [28]. As viral and bacterial treatments keep up, infectious diseases continue to be held at bay, and life expectancies push towards 100, protein aggregation or protein-lipid aggregation diseases, from Alzheimer’s to heart attacks, will be the primary cause of reduced quality of life in older patients.

I already mentioned domain size and chaperones as mechanisms to enable folding of large and complex structures. Several of the articles in this issue study chaperones. Yang proposes that some chaperones could act by binding to exposed sequences of proteins that would normally be prone to aggregation. Rotermann and coworkers analyze hydrophobic patches in GroEL to see how they can interact with proteins in a patterened way, and Semisotnov re-examines the question: GroEL: in the cavity, or not? In that context, work by Lorimer and Thirumalai is also of interest, as they propose a unfolding mechanisms whereby chaperone binding gives proteins a ‘second chance.’ by unfolding them and thus removing a misfold, yet caging also clearly helps [29,30].

Of course, many other molecules besides chaperones interact with proteins, and this leads us to the question of biological function. Ultimately, proteins do not fold to form pretty three-dimensional (or rather: less-than-three-dimensional) structures. If they fold at all, it is to fulfill a biological function. Several articles examine functional dynamics, or structure function relationships in more detail. Banhagi examines the role of small molecules as electron carriers to facilitate disulfide bridge formation. Vitamin C could be a major player – Linus Pauling would be glad to hear that it fulfills yet another important role. In the article by Linse and coworkers, folding and binding are combined, and the question becomes: is the mechanism for multi-unit assembly to fold first and then assemble, or *vice-versa* – or can both mechanisms be at play? Another assembly question is posed by Timsit: the ribosome is such a complex assembly of proteins and RNAs, that we have not even scratched the surface of how it can assemble to its final form, despite the availability of an X-ray structure now. How important are the disordered tails of some of the ribosomal proteins to help the RNA find its place? Surfaces of other biomolecules, and artificial surfaces, are also important binding sites for proteins. Stefani and coworkers discuss how surfaces can recruit proteins from 3-dimensional to 2-dimensional diffusion, thus enhancing aggregation. Such surfaces, be the anionic lipids, or collagen complexes, could enhance undesirable protein-protein interactions also, leading to aggregation. But not all is lost: Perret *et al*. discuss the possibility of nanoparticle surfaces acting as chaperones for proteins. In the work of Denny and coworkers, GAP (growth associated protein) is shown not to interact with the cytoskeleton directly, but rather with calmodulin, a calcium binding factor. They use elegant ^35^S labeling and cross linking experiments. Gehring and coworkers study a protein that binds more promiscuously, rather than less: BAG can interact with DNA directly, with heat shock proteins (hsp), and could couple transcription processes with post-translational quality control.

Speaking of coupling transcription and translation, Jan Biro discusses how the genetic code may not be so redundant after all. There are 64 base triplet combinations, but only 20 amino acids and STOP that need coding. In some cases up to four triplets code for the same amino acid. But as Biro discusses, different base triplets mean different RNA, and while protein function may not be affected by how an Ala residue was encoded, the RNA certainly is affected – and that in turn could encode information that makes it all the way to the protein, perhaps even in ways less obvious than controlling expression levels.

## 4. Conclusions

Clearly protein dynamics is a rich field. The answered questions are fascinating, unanswered ones abound, and even some old subjects – or perhaps especially those – can be quite controversial when examined in more detail. In this spirit, the present issue of IJMS will delve in a look at what is out there.
